# New biological insights into osteosarcoma—lessons from single cell sequencing studies

**DOI:** 10.1007/s10555-026-10336-z

**Published:** 2026-05-07

**Authors:** Julia I. Zehenter, Leo Kager, Sebastian K. Eder, Sabine Taschner-Mandl, Snežana Hinić

**Affiliations:** 1 St. Anna Children’s Cancer Research Institute, Zimmermannplatz 10, 1090 Vienna, Austria; 2 St. Anna Kinderspital, Kinderspitalgasse 6, 1090 Vienna, Austria

**Keywords:** Osteosarcoma, Tumor microenvironment, Single-cell RNA-seq, Single-nucleus RNA-seq, Single-cell transcriptomics

## Abstract

Osteosarcoma (OS), the most frequent primary malignant bone tumor in children and adolescents, is characterized by substantial inter- and intra-tumoral heterogeneity and an immunosuppressive tumor microenvironment (TME), constraining the efficacy of both standard chemotherapy and emerging immunotherapies. Recent advances in single-cell and single-nucleus RNA sequencing (scRNA-seq and snRNA-seq) have enabled high-resolution profiling of OS tumors, revealing diverse malignant, immune, and stromal cell populations. These studies have identified proliferative, inflammatory, and angiogenic tumor states, immunosuppressive myeloid subsets, exhausted T cells, and complex cell–cell communication networks that contribute to tumor progression and immune evasion. However, several challenges constrain the broader application of single-cell approaches in OS. The mineralized structure of bone tissue complicates dissociation into viable single cells, and the rarity of OS limits access to fresh specimens. Most existing datasets are based on small, heterogeneous cohorts and are generated using diverse protocols, which complicate data integration and comparison. Emerging methods are beginning to overcome these barriers. These include snRNA-seq for frozen and archival tissue, improved dissociation protocols for mineralized tumors, and integration with spatial transcriptomics to retain spatial context. Moving forward, combining single-cell transcriptomics with complementary modalities, such as immune repertoire analysis, chromatin accessibility profiling, and spatial proteo-genomics, combined with functional validation, will provide deeper insights into immune dynamics, regulatory mechanisms, and the cellular architecture of the OS TME. This review summarizes the current landscape of single-cell transcriptomics in OS and highlights methodological challenges in single-cell studies in OS tumors, recent biological insights, and their implication for immunotherapies.

## Introduction

Osteosarcoma (OS) is the most common primary malignant bone tumor, predominantly affecting children and adolescents [1]. It arises from mesenchymal cells [2], typically in the metaphysis of long bones [1] and is characterized by malignant osteoblasts that produce immature osteoid (unmineralized portion of the bone matrix), disrupting normal bone homeostasis [3]. OS represents a substantial clinical burden in the pediatric and adolescent population [3, 4]. Collectively, bone sarcomas account for approximately 4.7% of all cancers in children (0–14 years) and 7.8% in adolescents (15–19 years) [4–6]. In the USA, 750–900 new cases are diagnosed annually, including approximately 400 in individuals under 20 years of age [7]. In Europe, the total number of new cases across all age groups is estimated at around 1135 per year, with peak incidence in the 15–24 years of age group [4, 7–9].

Despite decades of research and treatment advances in the field of OS, including surgery, multi-agent chemotherapy, and targeted therapies [10], prognosis remains poor, especially for patients with primary metastatic or relapsed disease [1, 11, 12]. The 5-year survival rate for localized OS is approximately 60–70% [1, 13], but drops to below 30% for patients with metastatic OS [12]. The primary sites of metastases are the lungs and bones [12]. Spreads to other sites (e.g., the lymph nodes) are rare and associated with an even worse prognosis, with survival rates below 10% [14]. The unique genetic and biological heterogeneity of OSs seems to be one major obstacle hindering the identification of novel active systemic therapies for patients suffering from this devastating disease. No improvements in long-term systemic tumor control have been achieved since the introduction of standard chemotherapy regimens more than 40 years ago [1, 15, 16]. The landmark EURAMOS-1 trial underscored these challenges, demonstrating that even with large-scale international collaboration, intensifying or altering postoperative chemotherapy based on histological response did not significantly improve survival outcomes for poor responders [16–18].

OS is characterized by its complex and immunosuppressive tumor microenvironment (TME), composed of malignant, stromal, and immune cells [2, 3, 8, 9, 19, 20]. Furthermore, the heterogeneity, both inter- and intraindividual, in the tumor composition, contributes to immune evasion, therapy resistance, and variable patient treatment responses and outcomes [3, 4, 15, 21–23]. This heterogeneity includes different histological subtypes, diverse cellular populations, genomic and transcriptomic profiles of OS tumors, and cell function of these cell types [3, 4, 15, 21, 22]. High-resolution molecular profiling technologies are needed to better understand this complex and heterogeneous TME in OS. This enables the analysis of each cell type within the TME as well as their roles and interactions to better understand the mechanisms of therapy response and resistance [24, 25]. Traditional bulk transcriptomic approaches offer a low-cost option to survey the whole transcriptome. Bulk sample preparation is typically faster and more straightforward because it involves fewer time-critical and technically sensitive steps than single-cell technologies [26]. However, bulk measurements average signals across cells, resulting in lower data complexity and generally requiring a less complex analysis pipeline than single-cell transcriptomic approaches. Depending on the research question and experimental setup, bulk profiling may be sufficient to answer a given scientific question. While informative at a global level, bulk transcriptomic approaches average gene expression across heterogeneous cell populations and thus fail to resolve rare subpopulations or define precise cell states, making this one of the major limitations of bulk approaches [21, 26, 27]. In contrast, single-cell RNA sequencing (scRNA-seq) and single-nucleus RNA sequencing (snRNA-seq) offer the resolution necessary to analyze the OS landscape at the cellular level, enabling the identification of transcriptionally distinct cell types, inference of cellular developmental trajectories, and reconstruction of cell–cell communication networks [27–30].

Understanding the components of this complex TME and their interactions has a potential to ultimately lead to better-informed therapeutic choices in a personalized manner. Immune checkpoint inhibitor (ICI) therapy has shown promising results in many cancer types including melanoma [31], non-small cell lung cancer [32], and renal cell carcinoma [33]. However, its efficacy as monotherapy in sarcoma has been less successful [34–38]. Although OS has a high tumor mutational burden, even in the pediatric population of patients, this parameter on its own has been shown not to be a reliable predictive biomarker for a response to immunotherapy in all cancer types [39]. Additionally, the limited response to checkpoint blockade may also be in part due to an immunosuppressive TME [40] and many T cells having hallmarks of T cell exhaustion [21, 35, 41]. These observations underscore the need to better understand the OS TME to identify predictors of immune response and targets to improve efficacy of cellular immunotherapies.

This review aims to (i) summarize available osteosarcoma single-cell and single-nucleus transcriptomics research from year 2020 to 2026 and available datasets, (ii) synthesize core biological insights from single-cell transcriptomics studies and their implication for immunotherapies, (iii) evaluate technical and analytical challenges, and (iv) provide practical guidance where applicable.

## From bulk to single-cell transcriptomics in osteosarcoma

Bulk RNA sequencing (RNA-seq) has contributed substantially to our understanding of OS biology by capturing gene expression profiles across whole tumor samples [26, 42]. However, this approach averages signals across diverse cell populations, effectively masking the transcriptional complexity within the tumor and its microenvironment [43, 44]. As a result, rare but biologically significant subpopulations of cells may be overlooked, and interactions between distinct cell types cannot be resolved [21, 26, 27].

In contrast, scRNA-seq allows for the analysis of transcriptomes at the level of individual cells [45–47]. This enables a much deeper understanding of cellular heterogeneity and the dynamic organization of the TME. This is particularly relevant for OS, which is characterized by a highly heterogeneous TME composed of malignant osteoblastic or chondroblastic cells, stromal cells (such as fibroblasts and endothelial cells), and a diverse array of immune cells including macrophages, T cells, and dendritic cells, among other cell types [27–30, 48].

ScRNA-seq provides a powerful tool to identify (rare) cell subpopulations, discriminate between malignant and non-malignant cells, and examine heterogeneity within specific lineages [29, 49]. It also enables inference of functional states, lineage trajectories, and phenotypic plasticity [49–53]. In OS, this has facilitated the classification of proliferative, inflammatory, angiogenic, and stress-responsive tumor cell subtypes, as well as the discovery of immunosuppressive myeloid and exhausted T cell populations [26, 29, 30]. Beyond cell identification, scRNA-seq supports the reconstruction of cell–cell communication networks through ligand–receptor interaction analysis [53]. These approaches have been utilized to map key signaling pathways in OS, such as RANKL-RANK signaling between tumor cells and osteoclasts, VEGFA-VEGFR interactions promoting angiogenesis, and immune-modulatory crosstalk between cancer-associated fibroblasts and T cells [19, 27, 54, 55].

While bulk RNA-seq continues to serve as a valuable tool for broader transcriptomic comparisons, only single-cell approaches offer the resolution needed to investigate the full complexity of OS [26, 45]. As such, scRNA-seq and its emerging adaptations, including snRNA-seq, are poised to redefine our understanding of OS pathophysiology [14, 55, 56] and open new avenues for therapeutic intervention [5, 28, 55].

## Advancements and current landscape of single-cell transcriptomics in osteosarcoma—published studies and datasets

Despite the promise of single-cell technologies, the application of scRNA-seq and snRNA-seq in OS is still in its early stages. To date, only a limited number of studies have successfully profiled OS tumors at single-cell or single-nucleus resolution [5, 14, 54]. These efforts have been constrained by multiple technical and logistical barriers but have nonetheless begun to uncover the cellular complexity of this aggressive bone tumor.

Most studies conducted so far rely on small cohorts of often fewer than ten individuals and combine primary, relapsed, and metastatic samples [5, 30, 56]. One of the earliest studies in the single-cell OS transcriptomic field was conducted by Zhou et al. and laid the groundwork for the further studies on the intra-tumoral heterogeneity and TME of OS [5]. This study analyzed scRNA-seq data from 11 individuals with OS, with a combination of primary, relapsed, and metastatic tumors which consequently enabled a comprehensive look into TME. This foundational dataset has been widely reused and reanalyzed in subsequent studies to explore tumor heterogeneity, immune evasion, and cell–cell interactions [28, 54, 57]. The same year, another study in the field of OS transcriptomics was published. The authors aimed to systematically detect coordinated changes in metastatic transcript expression in primary bone cancers [58]. Their findings revealed that *MAPK7* acts as a master regulator of *MMP9* and signaling as a driver for primary bone cancer metastasis. In this study, 21 new OS tumor samples were analyzed, and single cells were extracted from circulating tumor cells from blood of patients with OS.

Other key contributions include studies by Liu et al. [14], He et al. [7], and Zheng et al. [54], who have either generated additional single-cell data or integrated new samples with previously published datasets. Liu et al. performed scRNA-seq on OS samples (tumor samples, adjacent tumor tissue, and lymph node) from six individuals [56], and in a subsequent study, reanalyzed previously published datasets while also adding samples from two additional individuals [14]. He et al. combined reanalysis of previously generated data with newly sequenced samples from four individuals, broadening the transcriptional landscape of OS [7]. Zheng et al. generated one of the largest single-cell OS datasets to date, comprising 27 tumor samples, of which ten were novel samples from ten individuals, and further strengthened their analysis by integrating spatial transcriptomics [54]. Cillo et al. employed a targeted approach, sequencing CD45^+^ sorted immune cells from four individuals with OS, enabling high-resolution profiling of the tumor-infiltrating immune compartment [59]. Recently, Shen et al. [60] have increased a pool of public datasets with 12 new treatment naïve datasets, including nine patients below 25 years of age and three patients of ages above 60. They have also performed TCR sequencing.

**Fig. 1:**
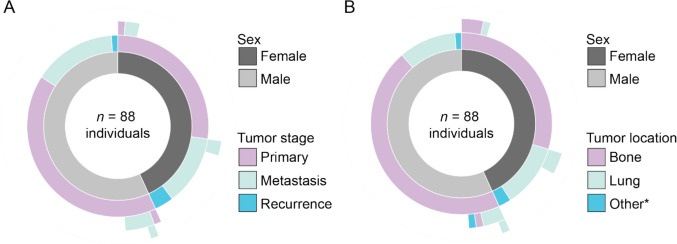
Characteristics of tumors included in the studies described in this review article. The data was available for 88 individuals out of 93. **A** Tumor stage including primary, metastatic, or recurrent tumors was quantified for female and male. **B** Tumor location including bone, lung, and other (asterisk denotes four tumors, one of each back, pelvis, paraspinal, and kidney) is described for female and male

Public resources have also contributed valuable datasets. The single-cell Pediatric Cancer Atlas (scPCA) [61] hosts scRNA-seq and snRNA-seq data from three OS studies (SCPCP000017; SCPCP000018 [62]; SCPCP000023), collectively encompassing 67 tumor samples from 56 individuals with OS. These publicly accessible datasets are a valuable resource for comparative and meta-analytic studies, but also highlight the scarcity of large, well-annotated OS single-cell datasets. A summary of key studies discussed in this review article, including patient numbers and sequencing methods, is presented in Table 1. Major limitations of these studies are overlapping and include small sample size, especially per different tumor stage (primary, recurrent, and metastatic tumors), lack of matched samples, often a pre-treatment bias is present, overall sparse metadata, and lack of information regarding therapy and survival outcomes, as well as a need for further functional validation. The overview of the available metadata for the single-cell transcriptomics datasets in OS studies is represented graphically in Fig. 1A and B.

**Table 1:** Overview of the number of individuals and osteosarcoma tumor samples from single-cell and single-nucleus RNA sequencing studies discussed in this review

Dataset ID	Source-study ID	Number of patients	Number of new tumor samples	Type of experiment^a^	Technology	Tumor stage	Sex	Median tumor onset age [range]	Treatment status known	Sequencing platform	Reference	Key biological insights
OS_public_01	scPCA Portal-SCPCP000017	20	27	sn	10Xv25prime	18 P, 9 met	12 M, 8 F	13 [4–21]^f^	No	NA	[48, 61]	Not discussed in this review for findings, but as a data resource
OS_public_02	scPCA Portal-SCPCP000018	10	11	sc	10Xv3.1	4 P, 1 R, 6 met	5 M, 5 F	18 [10–32]	No	Illumina NovaSeq 6000	[61, 62]	Not discussed in this review for findings, but as a data resource
OS_public_03	scPCA Portal-SCPCP000023	26	29^b^	sn^c^	10Xv3.1	15 P, 14 met	14 M, 12 F	14 [8–22]	18 yes^g^, 11 no	NA	[61]	Not discussed in this review for findings, but as a data resource
OS_public_04	GSE152048	11	11	sc	10X v2 and v3 3prime	7 P, 2 R, 2 met	NA	NA	Yes	Illumina NovaSeq 6000	[5]	- Osteoblastic OS cells can trans-differentiate from chondroblastic OS cells - TIGIT is highly expressed across tumors, and *in vitro* experiments show therapeutic potential of TIGIT blockade - Most metastatic lesions were mostly composed of osteoblastic cells - Pro-inflammatory *FABP4*+ macrophages are dominant in metastatic lesions
OS_public_05	GSE162454	6	6	sc	10Xv3 3prime	6 P	4 M, 2 F	17.5 [13–45]	No	Illumina NovaSeq 6000	[56]	- Malignant OS cells and CAFs promote angiogenesis via *VEGFA* - OS cells regulate osteolysis by inducing osteoclast differentiation through RANK-RANKL interaction - Most macrophages are *IFIT*+ (pro-inflammatory) and *TXNIP*+ (anti-inflammatory)
OS_public_06	GSE237070	2	6	sc	10Xv3	2 P	2 F	24 [14–34]	No	Illumina NovaSeq 6000	[14]	- Pseudotime analysis showed that osteoblast cells follow a trajectory that indicates their continuous activation and malignant transformation - *ETS2*/*IBSP* signaling axis promotes tumor cell invasion - Spatial transcriptomics showed CAFs and myeloid cells positioned as an immune barrier adjacent to OS cells
OS_public_07	^e^	4	4	sc	10Xv3.1 3prime	NA	NA	NA	NA	Illumina NovaSeq 6000	[7]	- Post-chemotherapy, *CD74*^+^ and *TPSB2*^+^ osteoblasts differentiated into more proliferative states and samples showed increased EMT, DNA repair, and G2M checkpoint pathways - *MMP9* is highly expressed in several osteoclast subtypes post-chemotherapy and *SPP1*+ CAFs involved in tumor progression and invasion are present
OS_public_08	GSE198896	4	4^d^	sc	10X v2 3prime and v1 5prime	1 P, 3 R	1 M, 3 F	NA	NA	NextSeq550 or NovaSeq 6000	[45]	- Relapsed tumors had a significantly higher immune infiltration compared to primary tumors - CD14+ CD16+ macrophages are identified as a primary driver of overall immune infiltration - High tumor mutational burden in OS correlated with the overall level of immune cell infiltration
OS_public_09	GitHub zhengxj1/A-Single-Cell-and-Spatially-Resolved-Atlas-of-Human-Osteosarcomas	10	10	sc	10Xv3 3prime	9 P, 1 R	4 M, 6 F	15.5 [11–67]	Yes^h^	Illumina NovaSeq 6000	[54]	- Spatial transcriptomics showed a spatial niche on the forefront of the necrotic zones that highly expressed *COL4A1* - Endothelial cells closely communicate with mesenchymal stem cells, pericytes, and CAFs via FN1 – (ITGA5 + ITGB1) as a key communication signal - Osteoclast maturation was tracked via pseudotime analysis and identified immature to mature osteoclast stages
OS_public_10	^e^	12	12	sc	GEXSCOPE Single Cell RNA Library Kit (Singleron)	NA	NA	NA	Yes^i^	Illumina NovaSeq 6000	[60]	Not discussed in this review for findings, but as a data resource

*scPCA Portal*, single-cell Pediatric Cancer Atlas; *scRNA-seq*, single-cell RNA sequencing; *v*, version; *sc*, single-cell; *sn*, single-nucleus; *M*, male; *F*, female; *P*, primary tumor; *R*, recurrent tumor; *met*, metastasis; *OS*, osteosarcoma; *CAFs*, cancer-associated fibroblasts; *TILs*, tumor-infiltrating lymphocytes; *EMT*, epithelial to mesenchymal transition

^a^Information about sample nature (fresh versus fresh-frozen) is not provided, but from single-cell versus single-nucleus it can be deduced that for single-cell studies, samples had to be most probably fresh, versus for single-nucleus studies, both fresh and fresh-frozen samples could be used

^b^Xenografts are not included in the count

^c^All tumor samples included are single-nucleus

^d^This study used sorted tumor-infiltrating lymphocytes and primary blood mononuclear cells

^e^Clinical information not publicly available

^f^For five tumors, there are no ages of tumor onset provided

^g^There is a note that the treatment status is known, but there is no more information provided in the metadata

^h^Only information about tumor being pre- or post-chemotherapy is provided, not the actual therapy regimen

^i^Information that the tumors did not receive pre-operative chemotherapy is only known

Taken together, these studies have built a foundational framework for characterizing the OS TME, highlighting key immune and stromal components, transcriptional subpopulations, and evidence of both intra- and inter-patient heterogeneity. Despite limitations in sample size and validation, they provide the first high-resolution snapshots of the TME, offering insight into the diverse populations of malignant, immune, and stromal cells that shape OS biology. Building upon the foundational datasets described above, recent work has provided detailed insights into the cellular composition of the OS TME as revealed by scRNA-seq, focusing on the major cell types and transcriptionally distinct subpopulations identified across recent datasets. Nevertheless, there are limitations to single-cell transcriptome studies in osteosarcoma that we will discuss below.

## Cellular composition of the osteosarcoma tumor microenvironment

Recent single-cell and single-nucleus transcriptomic studies have provided the first high-resolution maps of the OS TME. These analyses reveal that OS consists of a highly heterogeneous mix of malignant, immune, and stromal cells, with substantial intertumoral heterogeneity, which shows that even within a single tumor, cancer cells can have varied genomic profiles and biological functions [7, 29]. The composition of this TME can vary considerably between patients, adding another layer of complexity. Although the number of available studies remains limited, consistent patterns have begun to emerge. Notably, the OS TME is typically dominated by myeloid-derived immune cells, features multiple stromal populations, and harbors malignant cells of both osteoblastic and chondroblastic origin [5].

Additionally, the OS TME is often immunosuppressive, facilitating tumor progression and enabling immune evasion [21]. To build on these initial findings, several studies have reanalyzed existing scRNA-seq datasets to achieve deeper characterization of the OS microenvironment. A recent review article [9] collected findings from five major research articles in the field of OS TME, highlighting the studies by Zhou et al. [5] and Liu et al. [56].

**Fig. 2:**
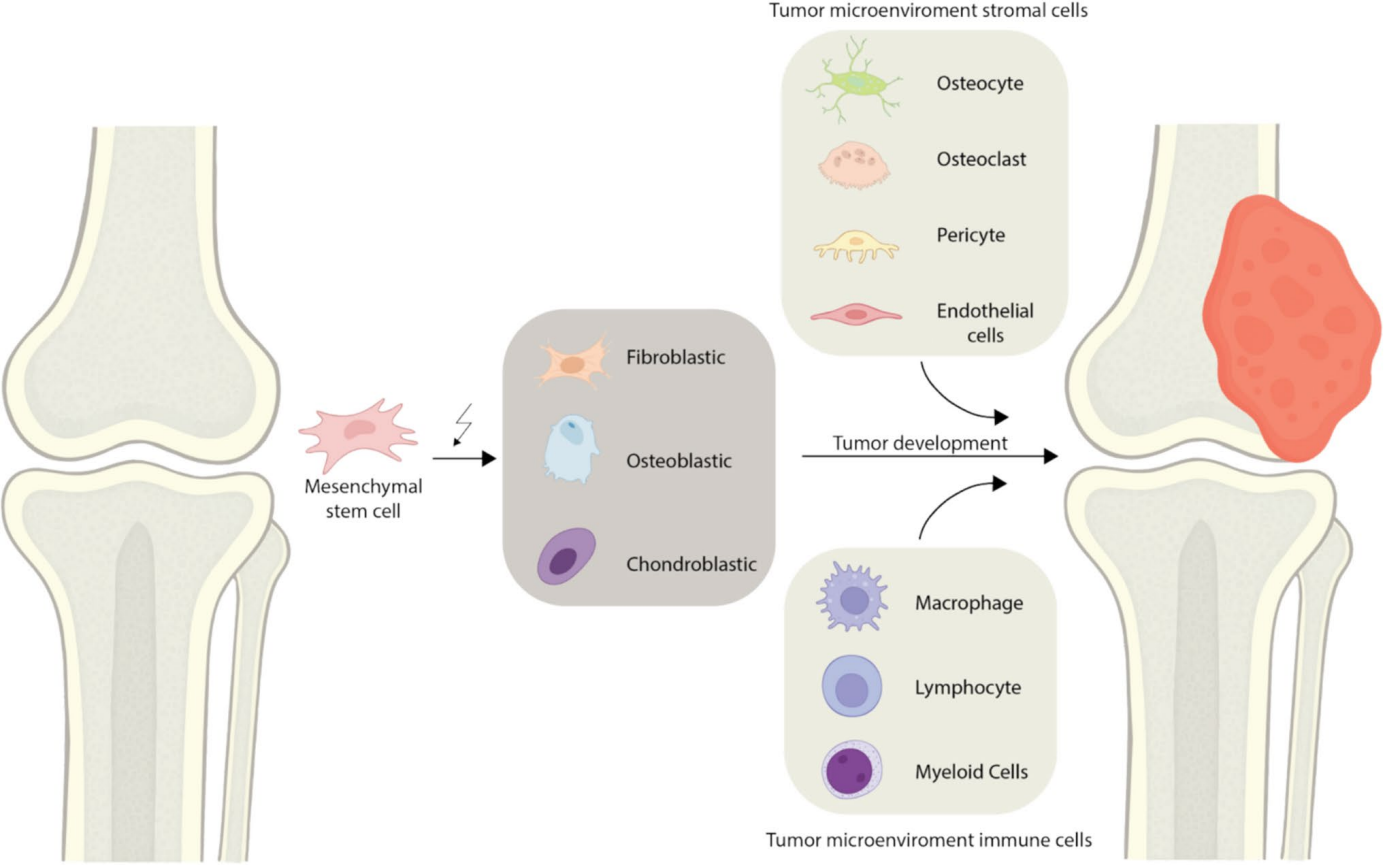
Schematic representation of the cellular diversity within the osteosarcoma (OS) tumor microenvironment. Osteosarcoma cells are of mesenchymal origin, arising from mesenchymal stem cells, and can differentiate along various stromal lineages, giving rise to a heterogeneous pool of malignant cells. In parallel, mesenchymal stem cells also contribute to the formation of non-malignant stromal populations, including osteocytes, osteoclasts, pericytes, and endothelial cells. The OS microenvironment is further shaped by infiltrating immune cells, such as macrophages, lymphocytes, and other cells of the myeloid lineage

Across these studies, the authors identified a shared set of major cellular clusters within the OS TME, including OS cells, osteoclasts, fibroblasts, mesenchymal stem cells, monocytes, macrophages, dendritic cells, tumor-infiltrating lymphocytes (TILs), B cells, natural killer (NK) cells, pericytes, endothelial cells, myoblasts, and proliferative cell populations (Fig. 2; [5, 29, 30, 56, 63]). Although some variability in abundance and subtype resolution exists between datasets, the overall cellular composition shows notable consistency. Differences in cell-type composition and abundance are evident when comparing single-cell and single-nucleus studies. Several studies have investigated this in different cancers, including lung cancer and hepatocellular carcinoma; however, this type of research has not been performed yet in OS. For example, a study in lung cancer showed that in the same tumor using scRNA-seq the TME was dominated by immune cells, whereas epithelial cells dominated in snRNA-seq datasets [64]. Inter-patient variability, sampling strategies, and other confounding variables can influence the observed results. However, across studies, a consistent trend emerges: single-cell approaches generally recover a higher proportion of immune cells than single‑nucleus methods [65]. Similar patterns have been observed in liver cancer and other tumor types [66, 67].

Cell type annotation in single-cell transcriptomic research in OS is primarily performed through the expression of canonical marker genes and further refined into distinct subclusters based on transcriptional profiles [5, 7, 55, 56]. Canonical marker genes help to define cellular populations in OS TME. Immune cells generally resemble those in other solid tumors and can be identified using established markers, e.g., *CD3D*, *CD3E*, and *CD3G* for T cells [68]. In contrast, developing reliable cell type marker sets to confidently distinguish malignant from non-malignant cells in the tumor and to resolve specific immune or stromal subtypes remains difficult.

Studies described in this review article have used different strategies, such as defining sets of marker genes for specific cell types and using clustering-based methods, to more confidently define cells in the TME. These strategies often rely on gene sets, both present and absent in specific cell types [5, 14, 56]. The markers used across studies are not always consistent, and sometimes not clearly specified, complicating the definition of canonical gene markers for each cell (sub)type in OS. Alternative approaches to aid cell type annotation employ clustering and whole transcriptome information to define cell subtypes [69]. Next to this, inferred copy-number alterations can be used to confirm malignant and non-malignant cell states [69]. Another strategy is to leverage a well-annotated reference dataset and perform label transfer to the dataset of interest [28]. However, such reference datasets are currently lacking for several OS-specific cell types. Generating a comprehensive dataset that includes normal tissue, OS tumor samples, and key TME components would be an important step toward establishing a robust reference for OS cell type annotation [70].

### Tumor cells in high-grade osteosarcomas

**Fig. 3:**
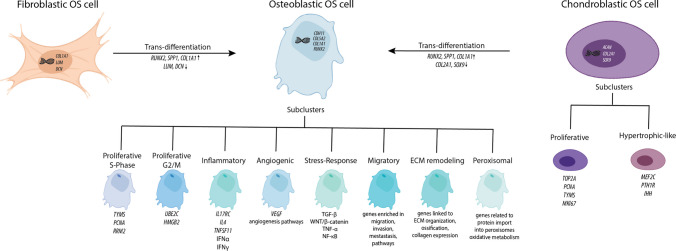
Transcriptional heterogeneity and plasticity of OS cells. Overview of fibroblastic, osteoblastic, and chondroblastic OS cell subtypes and their subclusters. Fibroblastic OS cells express *COL1A1*, *LUM*, and *DCN* and may trans-differentiate into osteoblastic OS cells (upregulation of *RUNX2*, *SPP1*, *COL1A1*; downregulation of *LUM*, *DCN*). Osteoblastic OS cells (defined by *COL1A1*, *COL5A2*, *CDH11*, *RUNX2*) include proliferative (S-phase: *TYMS*, *PCNA*, *RRM2*; G2/M: *UBE2C*, *HMGB2*), inflammatory, angiogenic, stress-response, migratory, ECM-remodeling, and peroxisomal subclusters. Chondroblastic OS cells (*ACAN*, *COL2A1*, *SOX9*) include proliferative (*TOP2A*, *PCNA*, *TYMS*, *MKI67*) and hypertrophic-like (*MEF2C*, *PTH1R*, *IHH*) subclusters. A transitional population shows a shift toward the osteoblastic state (upregulation of *RUNX2*, *SPP1*, *COL1A1*; downregulation of *COL2A1*, *SOX9*), supporting malignant lineage plasticity

Most osteosarcomas in young patients are of high-grade, and according to the current WHO classification, high-grade OSs are named not otherwise specified OS (NOS; World Health Organization Soft tissue and bone tumors, 5th edition) [71]. More than 80% of NOS are conventional osteosarcomas, which are further sub-classified based on the predominant histopathological components as osteoblastic, chondroblastic, or fibroblastic OS (Fig. 3). Rarer NOS subtypes include telangiectatic and small cell OS. OS cells show diverse transcriptional programs reflective of proliferative, inflammatory, angiogenic, and stress-response states. Osteoblastic OS cells are characterized by high expression of *COL1A1*, *CDH11*, and *RUNX2* [5], as well as *COL5A2* [72]. Current studies have identified multiple subclusters of osteoblastic OS cells, which include proliferative cells, identified by expression of specific cell cycle markers. Two subclusters were identified and are characterized by the high expression of cell cycle marker genes, specifically the S phase marker genes (*TYMS*, *PCNA*, and *RRM2*), and G2/M phase genes (*UBE2C* and *HMGB2*) [5]. Other studies have identified cellular clusters with high expression levels of *TOP2A* and assigned it to a proliferating cell label [7, 30, 56]. Moreover, multiple studies identified clusters of cells where specifically hallmark genes of angiogenesis and inflammation were observed to be highly expressed [73]. This includes genes involved in angiogenesis, IFN-α, and IFN-y pathways in one study [5] and high expression of the *IL17RC*, *IL4*, and *TNFSF11*, which are markers of inflammation, in another study [56]. Stress-related clusters are enriched for signaling associated with TGF-β, WNT/β-catenin, TNF-α, and NF-κB, all of which contribute to tumor progression, immune modulation, and epithelial-to-mesenchymal transition (EMT) [33, 55, 74]. Furthermore, genes responsible for migration and invasion, which are enriched in metastasis-related pathways and are related to a poorer survival response, were also identified in multiple studies [28, 55, 56, 74]. Additional subclusters involved in processes of ECM and protein import into the peroxisome matrix, ossification, and metabolism have also been described (Y. Liu et al., 2021).

Chondroblastic OS cells are characterized by the high expression level of markers such as *ACAN*, *COL2A1*, and *SOX9* [5]. Chondroblastic OS cells can, similarly to osteoblastic OS cells, be divided into subgroups. These subclusters include a proliferative cluster characterized by the expression of *TOP2A*, *PCNA*, *TYMS*, and *MKI67* [5]. Furthermore, hypertrophic-like clusters are also identified and defined by high expression of *MEF2C*, *PTH1R*, and *IHH*, and are enriched for signaling pathways such as IL-2/STAT5, Hedgehog, Notch, IL-6, JAK/STAT3, and inflammatory response genes [5]. Lastly, chondroblastic OS cells have also been found to trans-differentiate into osteoblastic OS cells, which suggests that the malignant osteoblastic cells could be originating from malignant chondroblastic OS cells. These cells were found in a separate cluster and exhibited an increased expression of *RUNX2*, *SPP1* (also known as osteopontin), and *COL1A1*, while downregulating *COL2A1* and *SOX9*, suggesting a phenotypic shift toward osteoblastic lineages [5].

Fibroblastic OS cells are malignant tumor cells that are classified based on their predominant tissue features resembling fibroblasts [75]. It has been found that malignant osteoblastic cells in the OS TME could trans-differentiate from malignant fibroblast cells [76]. Another study found that a cluster of OS cells followed a fibroblast-like trajectory [30]. Specific marker genes used to define fibroblasts in OS include *COL1A1*, *LUM*, and *DCN* [76].

### Immune cells

#### Myeloid compartment

Immune infiltration in OS is dominated by myeloid cells, with T and B lymphocytes present at lower frequencies [21, 57, 77]. The functional state of immune cells often reflects immunosuppression, exhaustion, or tumor-promoting roles [21, 57, 77]. Myeloid cells are one of the most commonly occurring cell types in the OS TME and account for about 35–53% of total cells, depending on the tumor type [30, 76]. These include macrophages, monocytes, dendritic cells, neutrophils, and mast cells.

Macrophages, referred to as tumor-associated macrophages (TAMs), present a range of functional states. First, CD14+CD16+ subsets often express HLA molecules involved in antigen presentation, while CD14+CD16− subsets are enriched in mTOR signaling and oxidative phosphorylation [59]. Next, M2-like macrophages express markers such as *TXNIP*, *MERTK* (involved in apoptotic cell clearance), *MRC1*, *STAB1*, and *CD163* (associated with anti-inflammatory macrophages) [56]. In contrast, M1-like macrophages show transcriptional signatures induced by IFN-α, IFN-γ, IL2/STAT5, IL6/JAK/STAT3, and inflammatory pathways [5, 56]. Furthermore, lipid metabolism-associated macrophages express *APOC1*, *APOE LGMN*, and *FABP5* [56]. Next, FABP4+ macrophages, identified in lung metastases, display M1-like pro-inflammatory properties [5]. Additionally, proliferative tissue-resident macrophages express *MCM5* and *MKI67* [56] and lastly, SPP1+ macrophages have been associated with angiogenesis, EMT, and tumor progression [14, 30].

Monocytes in the OS TME include classical CD14+ monocytes, which express *CD14*, *VCAN*, and S100A8/9/12 [56, 73], as well as non-classical CD16+ monocytes, with high *CD16*, *CDKN1C*, *LILRB2*, *ITGAL*, and *CX3CR1* expression and low expression of *CD14* [56]. Additionally, CXCL8+ monocytes express interleukin 8, a chemokine involved in neutrophil recruitment [5]. Furthermore, neutrophils are identified by *S100A8*, *S100A9*, and *G0S2* expression [5], while mast cells in OS express *FCER1A* (IgE receptor), *KIT*, and *HPGDS* [30, 56].

According to findings from Liu et al., within the OS TME three clusters of dendritic cells (DC) were identified [57]. These consist of the conventional type 1 DCs that are characterized by the expression of *CLEC9A* and *XCR1*. Conventional type 2 DCs are characterized by the expression of *CD1C*, *CLEC10A*, and *FCER1A* [30, 57]. Liu et al. identified the third cluster which they named mature regulatory DCs (mregDCs), which may be an OS tumor-associated DC population. These mregDCs are characterized by the expression of *CD83*, *CCR7*, and *LAMP3* [57]. Additional DC populations identified in OS TME include monocyte-derived CD14+ CD163+ DCs [5], CCR7+ activated DCs [5], and CD1C− CD141− DCs [7, 30].

#### Lymphoid compartment

Within the TME of OS, tumor-infiltrating lymphocytes (TILs) play an important role in the use of immunotherapies. In general, T cells include CD4+ T cells expressing *CD4* and cytotoxicity markers like *GZMA*, along with co-stimulatory molecules such as *TNFRSF14* (also known as HVEM), *TNFRSF25* (also known as DR3), and *ICOS* [5, 7, 56]. Subtypes of CD8+ T cells include early-stage, exhausted as well as cytotoxic CD8+ T cells. In general, CD8+ T cells express *CD8A* and cytotoxic markers *GZMB*, *GZMK*, and *GZMH*, as well as exhaustion markers *TIGIT* and *LAG3* [5, 7, 56, 59]. Moreover, CD4− CD8− T cells have also been observed in the OS TME [5, 56]. Regulatory T cells (Tregs) are identified by *FOXP3* and *IL2RA* expression and co-express immune-inhibitory receptors, such as *CTLA4* and *TIGIT* [5, 7, 56]. Natural killer T (NKT) cells co-express NK markers, e.g., *NKG7* and *GNLY*, while also expressing T cell markers such as *CD3D* and *CD8A* [5, 14]. They are activated as evidenced by *GZMB*, *GZMA*, and *IFNG* expression [5, 14]. Two additional T cell populations, proliferating and CXCL13+ T cells, have been identified by Zhou et al. [5].

B lymphocytes include follicular B cells (*MS4A1/CD20*, *CD79A/B*), naive B cells (CD27−, IGHD+), memory B cells (CD27+, IGHM−, low *IGHD/IGHM*, high *IGHG3*), and antibody-secreting plasma cells (*MZB1*, *SDC1/CD138*, *IGHG1*, *IGHG2*, *IGHA1*, *IGHA2*, *PRDM1*). Plasmacytoid dendritic cells (pDCs), found within B cell clusters, express *LILRA4*, *IL3RA*, *TCF4*, and *CLEC4C* [56].

Natural killer (NK) cells are identified by the expression of *NKG7* and *GNLY* [5, 7, 56]. NK cell subtypes include CD56bright CD16low and CD56dim CD16high, which are characterized by their distinctive expression patterns of the canonical cell markers *NCAM1* (also known as *CD56*) and *FCGR3A* (also known as *CD16*). The CD56bright CD16low subtype is a prevalent subtype in the OS TME [30]. NK cell migration into the OS TME is regulated in part by chemokine gradients, particularly those involving the receptor CXCR3 and its ligands CXCL9, CXCL10, and CXCL11 [78].

### Non-immune stromal cells

Stromal cells include all cells in the TME that form a supportive tissue and include fibroblasts (cancer-associated fibroblasts, CAFs), mesenchymal stem cells (MSCs), pericytes, osteoclasts, endothelial cells, and some rare cell types.

Fibroblasts (or more notably CAFs) are a specific type of fibroblast that promote tumor growth, metastasis, and therapeutic resistance [76]. It has been found that chemotherapy enhances CAF presence and their ability to modify the ECM, and they can also interact with endothelial cells and macrophages. One study described five subtypes of MSCs including matrix CAF (mCAF), inflammatory CAF (iCAF), myogenesis CAF (myoCAF), vascular CAF (vCAF), and pericytes [54]. While CAFs are not a subgroup of MSCs, there is evidence showing that under certain conditions, MSCs, which are non-hematopoietic precursor cells characterized by their multipotent nature, can differentiate into CAFs [79]. In general, MSCs represent a heterogeneous group of cells that highly express markers such as *CXCL12*, *SFRP2*, and *MME* (also known as *CD10*) [5]. Pericytes are distinguished by high expression levels of *ACTA2* and *RGS5* [5], and a higher proportion of pericytes has been linked to a more favorable prognosis in OS [55].

Osteoclasts are specialized multinucleated cells derived from the monocyte-macrophage lineage [30]. They play a crucial role in bone tissue resorption (i.e., osteolysis) and are characterized by high expression of *CTSK* and *MMP9* [5]. One study has identified subclusters of osteoclasts that included progenitor osteoclasts, mature osteoclasts, and hypofunctional and nonfunctional osteoclasts [56], while another classified them as *CTSK*-expressing osteoclasts, *LGALS9*-expressing osteoclasts, *CD74*-expressing osteoclasts, *CCL3A1*-expressing osteoclasts, and *HMGB2*-expressing osteoclasts [7]. A high expression level of *MMP9* has also been shown in *CTSK-*, *LGALS9-*, and *COL3A1-expressing* clusters, suggesting that *MMP9* can be a potential therapy target [7].

Endothelial cells are identified by *PECAM1* and *VWF* expression and are involved in angiogenesis and in tumor metastasis promotion [5]. Several subclusters of endothelial cells were identified in different studies including proliferative, venous, capillary, lymphatic, *LUM*-expressing, tip-like, and *PRRX1*-expressing endothelial cells [19, 30]. Specific subtypes of the endothelial cells, tip-like endothelial cells, that are *MCAM* positive are shown to be abundant in primary and metastatic tumor sites. *MCAM* is thought to promote metastasis, as it is also highly expressed in endothelial cells from metastatic lymph nodes [19]. It has been shown that after chemotherapy, endothelial cells show compromised differentiation ability [30].

Other cells present in the OS TME are some rare cell types, such as tissue stem cells [28] and myoblasts (mostly in lung metastases) that express *MYL1*, *MYLPF*, and *MYOG* [5, 29, 76].

Together, these findings illustrate the remarkable cellular diversity of the OS TME and highlight the power of single-cell transcriptomic technologies in uncovering novel subpopulations and functional states as well as cell communication pathways.

## Cell–cell communication and trans-differentiation of tumor cells in osteosarcoma tumor microenvironment

**Fig. 4:**
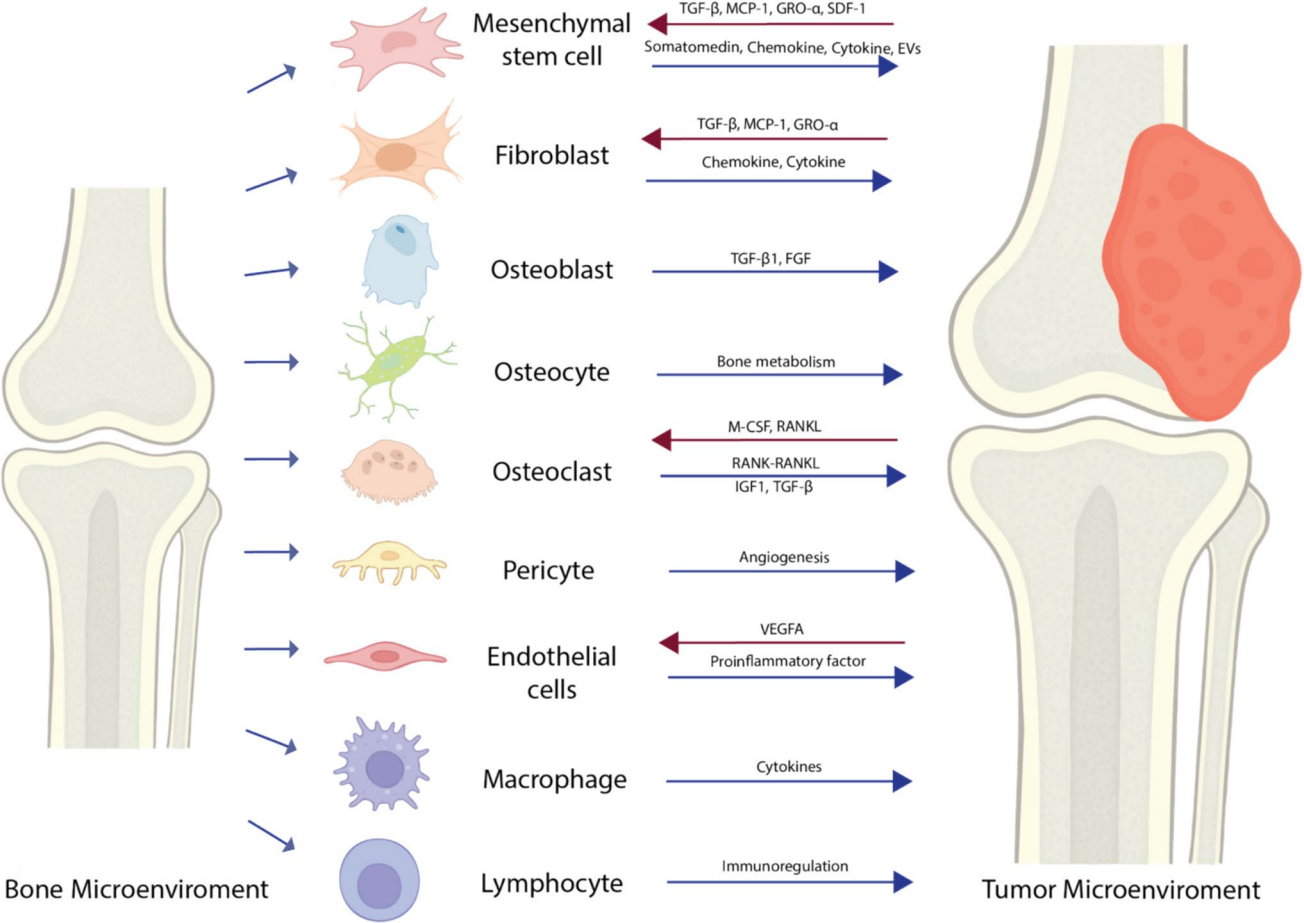
Schematic overview of the bone microenvironment highlighting key cell types involved in osteosarcoma (OS) progression. Mesenchymal stem cells, fibroblasts, osteoblasts, osteocytes, osteoclasts, pericytes, endothelial cells, macrophages, and lymphocytes all contribute to tumor development through distinct molecular signals. Representative factors secreted by each cell type are shown, including growth factors (e.g., IGF-1, FGF2), cytokines (e.g., IL-6, IL-10, TNF-α, RANKL), chemokines (e.g., MCP-1), and other regulatory molecules influencing angiogenesis, bone remodeling, inflammation, and immune modulation. These interactions collectively shape a dynamic and tumor-permissive microenvironment that facilitates OS progression and metastasis

The TME of human OS is not a static entity but rather a very dynamic and heterogeneous environment, in which malignant, stromal, and immune cells engage in constant bidirectional communication (Fig. 4). These interactions are mediated by receptor-ligand signaling, soluble factors, and extracellular matrix remodeling, all of which drive tumor growth, metastasis, and therapy resistance [3, 76].

### Cell–cell communication and functional states of cells in osteosarcoma

Figure 4 provides an integrated overview of these cellular interactions, illustrating how each cellular component of the OS bone microenvironment contributes distinct, but complementary signals that lead to the development of OS. Stromal elements (MSCs, fibroblasts), bone-resident cells (osteoblasts, osteocytes, osteoclasts), vascular cells (pericytes, endothelial cells), and immune infiltrates (macrophages, lymphocytes) all secrete growth factors, cytokines, and chemokines that converge to drive tumor proliferation, angiogenesis, osteolysis, and immune evasion. This network of communication highlights the cooperative nature of the OS TME in sustaining tumor progression and metastatic spread.

In more detail, osteoblasts and osteocytes contribute to tumor progression through growth factor signaling. Osteoblasts release TGF-β1 and FGF, whereas osteocytes regulate bone metabolism in ways that can indirectly support OS growth. Osteoclasts are central to tumor-bone crosstalk [5, 56, 80]. They are activated by M-CSF and RANKL secreted from OS cells and stromal cells. OS cells engage in crosstalk with osteoclasts through the *TNFSF11* (also known as RANKL)–*TNFRSF11A* (also known as RANK) axis, which particularly can drive osteolysis. This in turn provides both physical space and growth-promoting cues for tumor expansion and thereby promotes bone resorption. IGF1 and TGF-β released during bone resorption further feed back into tumor-promoting loops [5, 56].

OS cells and CAFs also interact with endothelial cells by secreting *VEGFA*, which activates angiogenesis through binding to *VEGF* receptors *FLT1* and *KDR* expressed on endothelial cells [56]. Furthermore, OS cells and CAFs communicate via different receptor-ligand pairs, such as those involving collagens and integrins that regulate cell adhesion, migration, and invasion [76]. CAFs can be reprogrammed by tumor cells, form physical barriers that hinder immune cell infiltration, and secrete factors like TGF-β, MCP-1, GRO-α, and additional chemokines and cytokines that remodel the ECM, collectively promoting tumor progression and metastasis [3, 30].

Other components in the OS TME also communicate actively [76]. Macrophages are highly abundant in the OS TME and exhibit diverse functional states. TAMs include both M1-like subsets with pro-inflammatory activity and M2-like subsets that promote immune suppression, angiogenesis, and extracellular matrix remodeling through cytokine secretion. Notably, a TAM subpopulation characterized by high *SPP1* expression has been linked to tumor progression, angiogenesis, and EMT [14, 20]. Finally, a crucial immune component in the OS TME are TILs. T cells often exhibit an exhausted phenotype due to prolonged antigen exposure, which impairs their cytotoxic function. These exhausted T cell populations are marked by the expression of inhibitory receptors such as *PDCD1* (also known as PD-1), *CTLA4*, *LAG3*, *ENTPD1* (also known as CD39), and *HAVCR2* (also known as TIM-3), and their presence is associated with immunosuppression and tumor progression in OS [21].

### Trans-differentiation of tumor cells in osteosarcoma

OS tumors show high mutational load, which is mainly driven by frequent somatic copy-number alterations (CNAs) and structural variants (SVs) [75, 76]. Next to that, OSs display chromothripsis [81], rather than being driven by a single common fusion oncoprotein [82]. ScRNA-seq studies allow inferring of the copy-number landscape on the level of single cells. Due to the genetics of OS, this is a particularly valuable approach in these tumors, as it aids in determining the inter- and intra-tumor heterogeneity on both transcriptional and genomic level between different cell populations in the TME [5, 30, 76]. This has proven useful in distinguishing malignant from non-malignant cells, as malignant OS cells show significant levels of CNAs [14]. One study showed more canonical CNAs present in primary and recurrent chondroblastic OS lesions when compared to osteoblastic OSs [5]. Nevertheless, common CNAs in OS that include amplifications on chromosomes 1, 3, 4, and 9, and deletions on chromosomes 2, 6, 10, 15, and 18 are found and are consistent with previously reported genomic CNAs [76]. When combined with the analysis of cellular developmental trajectories, the CNA landscape has been utilized to understand the trans-differentiation from osteoblastic to chondroblastic OS cells [5]. Some even suggest that malignant osteoblastic cells can be trans-differentiated from malignant fibroblast cells [5, 76]. Furthermore, trajectory analysis based on RNA velocity showed that osteoclasts differentiate from myeloid cells [76].

While these findings underscore the value of single-cell technologies in uncovering OS cell plasticity, their broader application is still constrained by substantial methodological and logistical challenges.

## Challenges in studying osteosarcoma at the single cell level

There are several limitations that impede studying OS on a single-cell level that stem from the heterogeneity of the currently available datasets and technical challenges in sample processing.

First, current cohort sizes remain small, and many studies rely on a few core datasets, such as that of Zhou et al. [5], or Liu et al. [56]. While this helps compensate for sample scarcity, it introduces batch effects and analytical inconsistencies due to variability in dissociation protocols, library preparation methods, and sequencing platforms [47, 83]. Furthermore, the field of OS single-cell transcriptomics faces clear limitations in terms of tumor stage coverage (e.g., primary and metastatic disease), access to fresh tissue, and the availability of longitudinal or matched control samples. Most existing studies combine different tumor states in a single analysis or focus on one group (i.e., metastatic samples), making it difficult to draw robust comparisons or infer temporal progression [5, 19, 28]. In addition to the study design and sampling constraints, a major limitation of the current OS single-cell literature is the general lack of functional validation of transcriptomic findings through *in vitro* or *in vivo* experiments [6, 19, 21, 28, 30]. Many observed cell states (e.g., from very specific immune cell types [57] to rare stromal cell types [28]), gene expression signatures (e.g., CD8 exhausted T cell gene signature [84]), or inferred pathways, such as those related to tumor–stroma crosstalk [7, 57], remain correlative and have not been mechanistically tested. Likewise, few studies have validated proposed biomarkers or transcriptional subtypes in clinical samples or linked them to treatment outcomes [28, 30].

To advance the field, several approaches can be utilized to overcome current limitations. First, increasing the number and genetic diversity of OS tumors profiled at single-cell resolution is critical to capturing the full landscape of cellular and genetic heterogeneity. This includes both scRNA-seq of fresh tissue and snRNA-seq of frozen or formalin-fixed paraffin-embedded (FFPE) samples [85]. Second, integrating sc/snRNA-seq datasets with orthogonal omics, including whole-genome or exome sequencing, copy number profiling, and DNA methylation, can enable molecular subtyping and help identify biomarkers of therapeutic relevance [86]. Third, developing analytical frameworks for harmonizing and integrating datasets, while accounting for batch effects and confounding variables, will be key to maximizing the utility of both newly generated and existing data [47, 83]. Defining optimal analysis strategies and harmonizing protocols remains challenging, as the best approach depends on the specific research question. Initiatives such as scPCA aim to standardize analysis across pediatric sc/snRNA-seq datasets in the scPCA portal [61]. While some sample-specific fine-tuning may still be necessary, following a consistent preprocessing workflow is essential. Key quality-control steps typically include assessing transcripts and genes detected per cell, mitochondrial gene content (as a marker of cellular stress), and ribosomal gene expression [87, 88]. Even at this stage, factors such as tissue-specific gene expression patterns and the choice of modality (single-cell vs. single-nucleus) should inform cutoff selection. Alternatively, a less stringent filtering strategy can be used, with low-quality cells being identified later during normalization, clustering, and differential gene expression analysis. Performing these steps at the individual-sample level before multi-sample integration is generally advisable.

For dataset integration, several tools, such as Seurat, Harmony, and scVI, are commonly used [89]. Comparing outputs from multiple methods can help determine the most suitable approach. Effective correction of confounders (e.g., sequencing batches) requires comprehensive metadata collection, typically including sex, age at diagnosis, tumor location, primary vs. metastatic status, treatment regimen, and sequencing-related information. Additional metadata such as tumor histology or grading can further improve interpretation, depending on the study aims. While basic good-practice principles in sc/snRNA-seq analysis are useful, no single pipeline fits all scenarios, as optimal choices depend on the sample type, RNA-seq modality, and research questions. The gap between transcriptomic discovery and biological or clinical relevance underscores the need for more integrative experimental pipelines and translational follow-up studies [5, 47, 83, 90]. For example, CRISPR perturbation screens in patient-derived OS organoids coupled with scRNA-seq could uncover regulators of tumor-intrinsic programs such as proliferation, EMT, and immune evasion. Spatial transcriptomics or multiplexed imaging could be suitable approaches to validate ligand–receptor interactions inferred from scRNA-seq, particularly within tumor–immune or tumor–stroma niches. In immune-focused studies, tracking TCR clonotypes before and after therapy can reveal clonal dynamics, persistence, and exhaustion states in response to immunomodulatory treatments.

Ultimately, progress in single-cell transcriptomics studies of OS will depend on large collaborative, multi-institutional efforts such as Fight OsteoSarcoma Through European Consortium (FOSTER) in Europe that prioritize high-quality standardized biobanking strategies, data sharing, and standardized pipelines [2, 91]. These efforts will not only help overcome the practical constraints of working with a rare tumor like OS but will also accelerate the discovery of clinically actionable insights through high-resolution single-cell analysis and enable longitudinal studies on larger sample size in the field of OS to be conducted.

## Biological and logistical barriers to sample acquisition and processing

Technical and logistical challenges stem primarily from the nature of bone tissue itself and the rarity of the disease [27, 28, 92]. Bone is characterized by its hardness, mineralized structure, and complex extracellular matrix (ECM) [93]. This makes the process of enzymatic tissue digestion and mechanical dissociation inherently challenging, which are crucial steps in preparing viable single-cell suspensions [27, 30]. Standard scRNA-seq protocols typically require fresh, intact, and viable cells, processed within hours of surgical resection to preserve RNA integrity and avoid dissociation-induced transcriptional artifacts [7, 56, 59]. Preparing single-cell suspensions from hard tissues such as bone, cartilage, or muscle often necessitates extended collagenase digestion, which can result in cellular damage, reduced yield, and transcriptomic bias [26, 27, 56, 59]. To generate high-quality single-cell suspensions, standard protocols in soft tissue tumors and other organs (e.g., lung, breast, or liver) typically use collagenase, DNase I, and occasionally hyaluronidase, followed by mechanical trituration and filtration. These workflows aim to optimize cell viability and RNA preservation [94–96]. Commercial solutions such as the Miltenyi Tumor Dissociation Kit, which combines optimized enzyme blends with gentleMACS™-based mechanical processing, have been used to improve digestion efficiency while reducing cellular stress [96–98]. In contrast, solid tumors like OS require more aggressive dissociation strategies, including prolonged digestion with collagenase II or IV, sometimes combined with dispase and DNase I, and additional mechanical disruption using tools like the gentleMACS™-dissociator [27, 56, 59, 95, 96, 99]. Protocols may also include decalcification or marrow depletion steps, yet yields remain highly variable depending on tumor composition, level of ossification, and necrosis.

Various approaches have been used across published studies. For instance, Zhou et al. [5] and He et al. [7] utilized the GEXSCOPETM tissue preservation and dissociation solutions from Singleron Biotechnologies, which enables rapid processing of fresh tissue within 30 min post-resection to ensure RNA integrity. There, tumors were minced into 1–2 mm fragments, enzymatically digested for 15 min at 37 °C, filtered, and subjected to red blood cell lysis [5, 7]. These protocols are streamlined and optimized, but rely on proprietary reagents, which may limit reproducibility and transparency. In contrast, Liu et al. [56] used a more conventional academic approach, digesting tumors with collagenase II for 45 min and following standard filtration and lysis protocols [56]. Despite differences in methodology, all studies achieved >80% cell viability prior to sequencing, demonstrating that scRNA-seq in OS is feasible. As noted earlier, reliance on different dissociation protocols contributes to batch effects and complicates data integration. Furthermore, in some cases, particularly when samples are necrotic, heavily calcified, or archived, researchers have opted for snRNA-seq instead, which bypasses the need for intact cells by isolating nuclei from frozen tissue [100]. While this method reduces dissociation-related artifacts and expands access to clinical samples, it often provides lower sensitivity for cytoplasmic transcripts [101].

Next to the challenging nature of OS tumors, the rarity of OS in pediatric patients and logistical considerations often make it difficult to obtain fresh tumor specimens [4, 19, 29, 59]. As a result, most available biobank samples are snap-frozen or FFPE, precluding standard scRNA-seq [4, 30, 59, 75, 99]. To circumvent this, snRNA-seq has emerged as a valuable alternative. By isolating nuclei rather than intact cells, it enables transcriptomic profiling of frozen and OCT-embedded tissues [100]. SnRNA-seq thus makes it possible to analyze archival OS specimens and access a broader range of clinical samples [15, 26, 99]. However, snRNA-seq comes with its own limitations compared to scRNA-seq, including reduced gene detection sensitivity and the absence of cytoplasmic transcripts. Additionally, certain activation states are underrepresented such as T cell exhaustion, IFN-γ response, cell types with low RNA content, or cytoplasmic marker reliance, including cytotoxic T cells, NK cells, and neutrophils [66, 75, 99].

## Technological advances enabling broader sample accessibility

There are several emerging technologies that are trying to expand the applicability of single-cell and single-nucleus transcriptomic profiling. Improvements in nuclear isolation techniques, low-input RNA library preparation, and optimized buffer systems are enhancing RNA quality and transcriptome coverage from frozen or fixed tissue, which are particularly valuable for rare cancer types with a limited number of available clinical samples [102, 103].

FFPE tissues are among the most widely available archival samples, but they present substantial challenges for transcriptomic analysis due to RNA fragmentation and chemical cross-linking introduced during fixation [30, 75, 99]. In response to these limitations, recent technological developments are expanding the applicability of single-cell approaches to previously inaccessible tissue types. One such improvement includes recent developments in scRNA-seq from FFPE samples, which made archival specimens accessible for transcriptomic studies [85, 104, 105]. Platforms such as 10× Genomics Fixed RNA Profiling, Parse Biosciences Evercode, and Illumina TruSeq FFPE have shown promise in recovering transcriptomic information from degraded RNA [105–107]. Although these methods still face challenges, such as lower gene recovery and the need for platform-specific computational pipelines, they offer new opportunities to utilize large, understudied FFPE biobank collections [75, 104, 108].

Beyond FFPE-specific approaches, additional innovations are broadening the overall accessibility and scalability of single-cell technologies. These advances aim to reduce technical complexity and financial barriers while improving data quality, such as combinatorial indexing, microfluidic-free approaches, and low-input library construction [109, 110]. Moreover, integrative frameworks that combine spatial transcriptomics with single-cell data (e.g., 10× Visium, NanoString CosMx, or Slide-seqV2 (111,112)) offer a way to preserve spatial context while capturing transcriptomic heterogeneity, an especially valuable feature for studying the tumor–stroma interface in OS [19]. In practical terms, this can be done, for example, by integrating spatial transcriptomics with sc/snRNA-seq via anchor [113], followed by co-detection [114] by indexing or imaging mass cytometry for protein-level validation [115]. Additionally, pre/post-therapy sampling with TCR sequencing can be added. Additionally, custom and ready-to-use bioinformatic pipelines for data analysis are being developed in parallel to these novel technologies (e.g., 10× Genomics Cell Ranger for sc/snRNA-seq data analysis; [116]). Collectively, these tissue-processing constraints, combined with the low prevalence of OS cases and limited biobank infrastructure (e.g., no standardized workflows for sample processing, storage infrastructure, etc.), contribute significantly to the relatively low number of publicly available OS scRNA-seq datasets [21, 30, 54].

It is also important to note that single-cell transcriptomic data can be generated using a range of platforms and library preparation chemistries, such as 10× Genomics 3 prime or 5 prime kits, Smart-seq2, or Drop-seq [117]. These technologies differ in transcript coverage, sensitivity, and the ability to capture specific features such as full-length transcripts, T cell receptor/B cell receptor sequences, or isoforms [66, 106, 107, 118, 119]. As a result, integrating or reusing publicly available datasets is not always straightforward, particularly when comparing data generated with different platforms, protocols, or sequencing depths. These technical differences must be carefully accounted for during analysis to ensure robust and biologically meaningful conclusions [47]. Nevertheless, overcoming these challenges, both in data generation and integration, is crucial for expanding the scale and scope of single-cell or single-nucleus RNA-seq research in OS and achieving a more comprehensive understanding of its complex biology.

## Implications for osteosarcoma immunotherapy

Despite increasing interest in immunotherapy, targeted therapeutic development remains challenging in OS due to the lack of recurrent actionable driver mutations, while attempts to leverage endogenous immune responses have not yet succeeded [32–34, 120]. Additionally, recent single-cell studies reveal that the OS TME is dominated by immunosuppressive myeloid cells (e.g., M2-like macrophages) and exhausted CD8+ T cells that express inhibitory markers like PD-1 [5, 21, 57, 77]. This suppressive environment likely hampers anti-tumor immunity and contributes to poor response rates to ICIs.

Notably, clinical trials testing anti-PD-1 monotherapy in OS have not demonstrated significant clinical benefit [34, 35], emphasizing the need for combination strategies. Emerging single-cell analyses suggest gene signatures of T cell exhaustion [21, 84, 121] and immunosuppressive ligand–receptor interactions as potential predictors of immune evasion. To improve the response to immunotherapy, future efforts involve combining TME-modulating agents with ICIs or cell-based therapies. For instance, the ongoing trial of Vactosertib (NCT05588648) targets the TGF-β receptor to dismantle the immunosuppressive stroma. Similarly, newer approaches are finally addressing previously “undruggable” drivers; the phase 2 OSTEOMYC trial (NCT06650514) is currently evaluating OMO-103, a first-in-class mini-protein inhibitor of the MYC oncogene, which is frequently amplified in aggressive OS.

Ongoing clinical trials are also evaluating complementary immunotherapeutic approaches, such as CAR-T cell therapies targeting GD2 or B7-H3. However, the recent suspension of the GD2-targeted CAR-T trial (NCT04539366) underscores the persistent challenges regarding safety and “on-target, off-tumor” toxicities in OS. Beyond primary immunotherapy, multi-kinase inhibitors like Cabozantinib (NCT02243605) continue to be explored. Building on earlier signals of efficacy, the large-scale AOST2032 trial (NCT05691478) is now investigating the addition of Cabozantinib to standard MAP chemotherapy in newly diagnosed patients.

A recent review highlighted that the dense immunosuppressive stroma and heterogeneous antigen expression in OS pose significant barriers to these therapies, necessitating combinations with macrophage-modulating agents or cytokines to enhance efficacy [121]. These evolving strategies, while promising, require validation in rigorously designed clinical trials that incorporate longitudinal immune monitoring and molecular profiling to identify predictive biomarkers and mechanistic correlates of response. Multi-omics studies including sc/snRNA-seq will help to identify new targetable cell–cell communication axes, T-cell receptor repertoires, and (patient-specific) landscapes of tumor-associated antigens to design better cellular therapies against osteosarcoma.

## Future directions and conclusion

Single-cell and single-nucleus transcriptomics have transformed our ability to dissect the OS TME, revealing unprecedented cellular heterogeneity, immune suppression, and dynamic cell–cell interactions. However, despite the emergence of detailed cellular maps and novel subtype classifications, key gaps remain that hinder clinical translation.

A major limitation across current studies is the lack of functional validation. While single-cell studies have identified candidate gene signatures, inferred differentiation trajectories, and mapped ligand–receptor interactions, these findings are largely correlative. Experimental validation using orthogonal methods, such as lineage tracing [122], CRISPR-based perturbation [123], or organoid co-culture models [124], remains essential to confirm the biological relevance of transcriptionally defined cell states, gene signatures, and inferred cell–cell interactions. Functional studies are especially needed to investigate mechanisms underlying immune evasion, such as T cell exhaustion [125], and to test therapeutic strategies aimed at overcoming immunosuppression or chemoresistance.

Additionally, most available datasets are derived from small, heterogeneous patient cohorts using diverse platforms and protocols. This not only limits statistical power but also introduces technical batch effects that complicate cross-study comparisons. To address this, future work must prioritize harmonization of sample processing, sequencing protocols, and data analysis pipelines. Public resources such as the scPCA provide a starting point, but collaborative initiatives focused specifically on OS are needed to scale up sample size and improve annotation depth. Increasing cohort sizes is critical for capturing the full spectrum of genetic and transcriptomic heterogeneity present in this rare and aggressive tumor. Larger, more genetically diverse datasets would allow researchers to identify underlying genetic differences that drive observed transcriptional programs and better stratify patients into molecularly defined subgroups. This, in turn, may improve prognostic assessments and facilitate more personalized therapeutic approaches.

Another underexplored frontier is the spatial architecture of the OS TME. Integration of sc/snRNA-seq with spatial transcriptomics [126] and proteo-genomics [127] will be crucial for understanding the topographical organization of malignant and stromal niches, as well as the physical barriers to immune infiltration. Such approaches will help reveal not just what cell states exist, but where they are localized and how they interact. In parallel, future studies should employ multi-omics approaches at the single-cell level to achieve a more comprehensive understanding of OS biology. Integrating transcriptomic data with T cell receptor profiling, chromatin accessibility (e.g., ATAC-seq [128]), and spatially resolved gene or protein expression [129, 130] can uncover clonal dynamics of immune cells, elucidate gene regulatory mechanisms, and map the physical organization of tumor–immune interactions. These approaches will help define the developmental origins of OS subtypes, reveal drivers of immune evasion and metastasis, and identify context-specific therapeutic vulnerabilities. Furthermore, applying these techniques longitudinally, especially in pre- and post-treatment samples, could illuminate mechanisms of therapy resistance or immune escape.

Developing robust prognostic and predictive models based on single-cell data will require concerted efforts in data harmonization, clinical annotation, and computational analysis. Collaborative, multi-institutional initiatives are needed to support biobanking of high-quality, annotated samples, enable standardized workflows, and foster open data sharing. Given the rarity of OS, such collective action will be essential to generate reproducible insights and translate them into improved outcomes for patients.

## Data Availability

No new datasets were generated or analyzed in this study.
